# Fever of unknown origin (FUO): which are the factors influencing the final diagnosis? A 2005–2015 systematic review

**DOI:** 10.1186/s12879-019-4285-8

**Published:** 2019-07-22

**Authors:** Francesco Maria Fusco, Raffaella Pisapia, Salvatore Nardiello, Stefano Domenico Cicala, Giovanni Battista Gaeta, Giuseppina Brancaccio

**Affiliations:** 1 Infectious Diseases Unit 1, S. Maria Annunziata Hospital, Central Tuscany Health Unit, Via dell’Antella 54, 50012 Bagno a Ripoli, FI Italy; 2 Epidemiology and Pre-clinical Research Department, National Institute for Infectious Diseases “L. Spallanzani”, Rome, Italy; 3 Infectious Diseases and Viral Hepatitis, Department of Mental and Physical Health and Preventive Medicine, University of Campania “Luigi Vanvitelli”, Naples, Italy; 4 Infectious Diseases, Azienda Ospedaliera, University of Campania “Luigi Vanvitelli”, Naples, Italy; 5 Infectious Diseases, Department of Mental and Physical Health and Preventive Medicine, University of Campania “Luigi Vanvitelli”, Naples, Italy; 6 Department of Molecular Medicine, Infectious Diseases, University of Padua, Padua, Italy

**Keywords:** Fever of unknown origin, Diagnostic outcomes, Infectious diseases, Neoplasms, Noninfectious inflammatory diseases

## Abstract

**Background:**

The differential diagnosis of Fever of Unknown Origin (FUO) is very extensive, and includes infectious diseases (ID), neoplasms and noninfectious inflammatory diseases (NIID). Many FUO remain undiagnosed. Factors influencing the final diagnosis of FUO are unclear.

**Methods:**

To identify factors associated with FUO diagnostic categories, we performed a systematic review of classical FUO case-series published in 2005–2015 and including patients from 2000. Moreover, to explore changing over time, we compared these case-series with those published in 1995–2004.

**Results:**

Eighteen case-series, including 3164 patients, were included. ID were diagnosed in 37.8% of patients, NIID in 20.9%, and neoplasm in 11.6%, FUO were undiagnosed in 23.2%. NIIDs significantly increased over time. An association exists between study country income level and ID (increasing when the income decreases) and undiagnosed FUO (increasing when the income increases); even if not significant, the use of a pre-defined Minimal Diagnostic Work-up to qualify a fever as FUO seems to correlate with a lower prevalence of infections and a higher prevalence of undiagnosed FUO. The multivariate regression analysis shows significant association between geographic area, with ID being more frequent in Asia and Europe having the higher prevalence of undiagnosed FUO. Significant associations were found with model of study and FUO defining criteria, also.

**Conclusions:**

Despite advances in diagnostics, FUO still remains a challenge, with ID still representing the first cause. The main factors influencing the diagnostic categories are the income and the geographic position of the study country.

## Background

Fever of unknown origin (FUO) was originally defined by Petersdorf and Beeson [1] as an illness of more than 3 weeks’ duration, with fever greater than 38.3 °C (101 °F) on several occasions, the cause of which is uncertain after 1 week of in-hospital investigations. To meet the evolution of diagnostic capabilities, some modifications in the definition of FUO occurred through the years: in 1991, Durak and Street proposed that there be a distinction between classical FUO and three other types, namely nosocomial, neutropenic and HIV-associated FUO; moreover, they reduced the duration of investigation, before defining a FUO, to at least 3 days in hospital or at least 3 outpatient visits [2]. In recent years, some authors [3–6] proposed to change the quantitative criterion (diagnosis uncertain after 1 week or 3 days of investigation) with the qualitative requirement that fever remained undiagnosed after a minimal diagnostic work-up had been performed; however, investigations that should be included in the work-up remain a matter of debate.

The differential diagnosis of FUO is the most wide-ranging in medicine, since more than 200 conditions have been identified as the cause of FUO [7]. Infectious diseases (ID), neoplasms and non-infectious inflammatory diseases (NIID) are the main categories of diseases causing FUO. However, despite recent advances in medicine, about a quarter of FUO remains undiagnosed [7, 8].

In published case-series, the spectrum of diseases causing FUO is very different, due to several factors still poorly explored. Among these factors, geographic prevalence patterns, the patient’s age, and the gap between the investigative resources of developing and developed countries may have a great significance. Clinical profile of FUO could have changed over time, in consideration of advances in diagnostic techniques, evolving socioeconomic status of the countries, development of new broad-spectrum pharmaceuticals, the emergence of new diseases and the attitude of physicians. The different methodologies among case-series (definition of FUO, retrospective or prospective model, use and composition of a minimal diagnostic work-up), may contribute to determine the final distribution of various causes of fever and the prevalence of FUO remaining without a diagnosis.

In order to identify the main factors influencing the clinical spectrum of FUO, we performed a systematic review of classical FUO case series including patients from 2000 and published in 2005–2015. Moreover, to describe the FUO characteristics over time, we compared our results with those of a systematic review on the same topic previously performed by our group, that analysed case-series published from 1994 to 2004 including patients from 1972 to 2002 [8].

## Methods

This paper has been written according to PRISMA checklist for the reporting of systematic reviews and meta-analysis [9]. We performed a systematic review with the following inclusion criteria: all case-series about classical FUO, published in 2005–2015, and having a starting data of patients’ inclusion from 2000 onwards.

### Search strategy

Eligible studies for this systematic review were identified through searches of PubMed for articles published from January 2005, to May 2015, by use of the following terms “fever of unknown origin” or “FUO” or “Pyrexia of unknown origin” or “PUO”, both as MeSH and Search terms. Articles resulting from these searches and relevant eligible studies cited in those articles were reviewed. Articles published in English were included.

### Data selection

The analysis of results emerged from this search strategy was conducted independently by two researchers (FMF, RP), on the basis of the title, abstract and full text were appropriate. After the selection of eligible studies, data from selected case-series were extracted manually by 3 researchers, independently. Later in the data analysis, all authors analysed extracted data to clarify some slight differences emerged in the data extraction. We analysed data about patients’ characteristics (M/F ratio, mean age), the model of study, the criteria used for defining FUO, the geographic area and the study country income classification, the use of a minimal diagnostic work-up, and the final diagnosis.

The quality of selected study was assessed according to tool proposed by Mudar at al [10], limited to questions 1,2,3,7 and 8, since questions 4–6 are relevant to cases of adverse drug events.

### Statistical methodology

Statistical differences among frequencies were calculated with the Mantel-Haenszel Chi-squared test, using an alpha significance level of 0.05. In order to identify the factors influencing the clinical spectrum of FUO, a logistic regression was performed using each final diagnostic category as outcome variable and significant variables at univariate analysis as covariate. The results derived from the logistic regression were represented by a forest plot, in order to graphically show the odds estimates with the relative confidence intervals, highlighting the type of association between the variables and outcomes. Data were analysed using the SAS software, version 9.3.

## Results

### Results of data selection

**Fig. 1:**
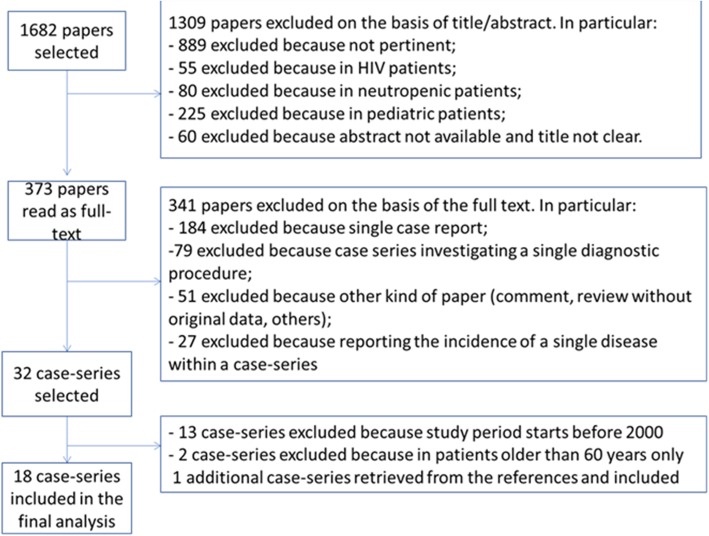
Flow Chart for the selection of 2006–2014 case-series about FUO

The search approach produced 1682 results. Among these, 1309 were excluded based on the title or abstract: 889 were not pertinent, 55 were about HIV patients, 80 described FUO in neutropenic patients, 225 among paediatric populations, finally in 60 cases the title was not clear and the abstract not available. The remaining 373 papers have been read as full text, and 341 have been excluded: 184 were single case reports, 79 were case series investigating the diagnostic value of a single procedure, 27 were papers describing the incidence of a single disease among a case-series, 51 were comments or reviews without original data. Among the remaining 32 case-series, 13 have been excluded because the study period started before 2000, and 2 because they included geriatric patients only. An additional case-series has been retrieved among the bibliography of selected papers, therefore we finally included 18 case series [3, 11–27]. Figure 1 shows the complete flow chart for study selection.

### Characteristics of selected studies

A total of 18 case-series, including 3164 patients, were analysed. General characteristics of case-series and patients included are summarized in Table 1. Quality of studies resulted good, with scores ranging from 3 to 5 (range 0–5). Case-series were from Asia or Europe; Middle East was the most represented among WHO regions. No case-series from Africa and from the Americas matched inclusion criteria. According to the World Bank income classification [28], 8 case-series were from upper-medium income, 6 from high income areas, and the remaining 4 are from lower-medium income areas. Most studies were retrospective, and most of them defined FUO according to Durak and Street definition; in 5 studies the criteria for defining FUO were personal, mostly including a qualitative assessment after a minimal diagnostic work-up, rather than a quantitative, time-dependent definition only. The enrolment period ranged from 1 to 10 years. Each case-series included at least 52 patients (range 52–997), 49.6% of patients were male, aged from 10 to 94 years.

**Table 1:** General characteristics of the selected case-series

Ref	First Author	Year	Quality assessment (range 0–5)	Country	Geographical area	Country income classification	Model of study ^a^	FUO criteria ^b^	Study period	Number of patients	M/F ratio	Mean age (range)	Minimal diagnostic work-up
[11]	Yu	2014	3	China	Far East	Upper- medium	1	1	2010–2011	107	54/53	48 (15–94)	No
[12]	Mir	2014	5	India	Southern Asia	Lower- medium	2	1	2010–2012	91	62/29	NA (16–80)	No
[13]	Naito	2013	4	Japan	Far East	High	1	1	2011	121	69/52	59 (19–94)	No
[14]	Robine	2014	5	France	Europe	High	1	1	2002–2012	103	54/49	57 (19–84)	Yes
[15]	Vanderschueren	2014	5	Belgium	Europe	High	1	3	2000–2010	436	NA	50 (NA)	Yes
[16]	Alavi	2013	3	Iran	Middle East	Upper -medium	1	3	2007–2011	106	57/49	50 (18–76)	NA
[17]	Mahmood	2013	3	Pakistan	Southern Asia	Lower- medium	1	1	2006–2011	205	111/94	38 (NA)	No
[18]	Shi	2013	5	China	Far East	Upper -medium	1	2	2004–2010	997	466/531	43 (14–85)	No
[19]	Mete	2012	3	Turkey	Middle East	Upper -medium	1	1	2001–2009	100	53/47	45 (16–82)	Yes^c^
[20]	Pedersen	2012	4	Denmark	Europe	High	1	1	2005–2010	52	36/16	48 (34–64)	No
[21]	Ali-Eldin	2011	3	Egypt	Middle East	Lower- medium	2	3	2009–2010	93	45/48	34 (NA)	No
[22]	Bandyopadhyay	2011	4	India	Southern Asia	Lower- medium	2	1	2008–2009	164	82/82	42 (NA)	No
[23]	Adil Khalil	2010	4	Iraq	Middle East	Upper -medium	2	1	2002–2009	55	27/28	43 (10–76)	No
[24]	Hu	2008	3	China	Far East	Upper -medium	NA	2	2002–2003	142	69/73	49 (14–81)	Yes^c^
[25]	Kucukardali	2007	4	Turkey	Middle East	Upper -medium	2	1	2003–2004	154	83/71	42 (17–75)	No
[3]	Bleeker-Rovers	2007	5	Netherland	Europe	High	2	3	2003–2005	73	33/40	54 (26–87)	Yes
[26]	Colpan	2007	4	Turkey	Middle East	Upper -medium	NA	3	2001–2004	71	40/31	42 (16–80)	No
[27]	Chin	2006	5	Taiwan	Far East	High	2	1	2001–2002	94	57/37	56 (18–86)	Yes

^a^ Model of Study: 1, Retrospective; 2, Prospective; ^b^ FUO criteria: 1, Durak and Street; 2, Petersdorf and Beeson; 3, personal criteria; ^c^ Diagnostic work-up performed, but not detailed; *NA* Not Available

Comparing these case-series (new case-series) with our previous systematic review, including 11 case series (old case-series) published in 1995–2004, no differences were present in geographical distribution, with most of case-series performed in Asia in both cases. Differences were present regarding the gender distribution (males 56% in old case-series and 49.6% in new case-series, *p* = 0,003) and the mean age of patients (40.6 in old case-series to 45.8 in new ones). Most studies were retrospective in both series. On the contrary, the definition criteria of FUO by Durak and Street was more frequently adopted among new case-series (11 out of 18) compared with old case-series (3 out of 11).

### Use of a minimal diagnostic work-up to qualify a fever as FUO

**Fig. 2:**
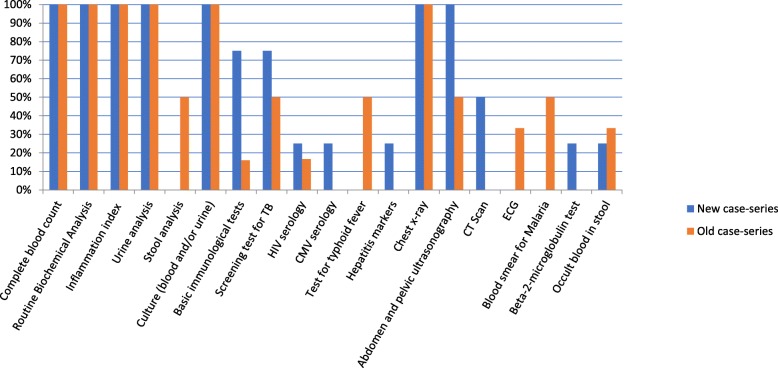
Comparison of tests and procedures included in a “Minimal Diagnostic Work-up” performed before qualifying a fever as FUO in new (2006–2014) and old (1995–2004) case-series

A predefined minimal diagnostic work-up to qualify a fever as FUO was adopted in 6 series and described in detail in 4. In general, complete blood count, routine haematochemical tests, inflammatory indexes, including C-reactive protein and/or Erythrocyte Sedimentation Rate, urine analysis, blood and urine cultures, chest x-ray and abdominal and pelvic ultrasonography were included. Four case-series performing the minimal diagnostic work-up were from high-income countries and two, in which it was reported but not described, were from an upper-medium income country. The routine use of a minimal diagnostic work-up decreased from 55% in old case-series (6 out of 11) to 33% in new case-series (6 out of 18). A comparison between the tests and procedures included in the new and old case-series is reported in Fig. 2. Instrumental diagnostic procedures, such as TC scan and ultra-sonography, were more frequently performed in the new case-series, as immunological tests and screening tests for tuberculosis. Instrumental procedures were more frequently included in the minimal diagnostic work-up in studies coming from high-income countries; as expected, some specific tests for tropical diseases (such as malaria and typhoid fever tests) were included in studies coming from medium-lower income countries.

### Diagnostic outcomes

Final diagnoses in the 18 case-series are reported in Table 2. Overall, infections were the most represented diagnosis (37,8%), followed by NIID (20,9%), neoplasm (11,6%), other diseases (6,5%); the diagnosis remained unknown in 23,2% of cases.

**Table 2:** Diagnostic categories in 18 case-series 2006–2014

Ref	First Author	Year	Number of patients	Infectious Diseases (%)	Neoplasm (%)	NIID (%)	Other (%)	No diagnosis (%)
[11]	Yu	2014	107	29.9	17.8	16.8	14.0	21.5
[12]	Mir	2014	91	44.0	12.0	12.0	5.0	27.0
[13]	Naito	2013	121	23.1	10.7	30.6	12.4	23.1
[14]	Robine	2014	103	23.5	2.9	30.1	4.9	50.5
[15]	Vanderschueren	2014	436	17.0	11.0	24.0	9.9	39.0
[16]	Alavi	2013	106	44.3	12.3	17.9	10.4	15.0
[17]	Mahmood	2013	205	48.8	12.7	18.6	3.4	16.6
[18]	Shi	2013	997	48.0	7.9	16.9	7.1	16.6
[19]	Mete	2012	100	26.0	14.0	38.0	2.0	20.0
[20]	Pedersen	2012	52	32.0	13.0	55.0	0.0	21.0
[21]	Ali-Eldin	2011	93	41.9	30.1	15.0	0.0	12.9
[22]	Bandyopadhyay	2011	164	54.9	22.0	11.0	0.0	12.2
[23]	Rami	2010	55	32.7	16.4	25.4	5.4	20.0
[24]	Hu	2008	142	35.9	12.7	32.4	4.9	14.9
[25]	Kucukardali	2007	154	34.4	14.3	30.5	5.2	15.6
[3]	Bleeker-Rovers	2007	73	16.0	7.0	22.0	4.0	51.0
[26]	Colpan	2007	71	45.1	14.1	26.8	5.6	8.5
[27]	Chin	2006	94	57.4	8.5	7.4	8.5	18.1
	Total		3164	37.8	11.6	20.9	6.5	23.2

Table 3 shows the 10 most frequent diagnoses for infectious diseases, neoplasm and NIID. Among infectious diseases, mycobacterial infections (mainly extra-pulmonary tuberculosis) were predominant, followed by endocarditis and abscesses; haematological malignancies represented 58% of neoplasm; diagnoses among NIID were more heterogeneous, with Adult Onset Still’s Disease, Systemic Lupus Erythematosus and vasculitis representing the most frequent.

**Table 3:** 10 most frequent diagnosis for infectious diseases, neoplasm and NIID in 18 case-series 2006–2014

Diagnosis	N° (%)	N° of case-series including the diagnosis
Infectious Diseases (out of 1197 cases from 18 case-series where details are available)		
Mycobacterial diseases	440 (36.8%)	17
Endocarditis	119 (9.9%)	15
Brucellosis	58 (4.8%)	9
Internal abscesses	49 (4.1%)	11
Salmonellosis	43 (3.6%)	9
CMV infections	43 (3.6%)	7
Urinary tract infections	40 (3.3%)	11
Sepsis	20 (1.7%)	4
HIV/AIDS	20 (1.7%)	4
Osteoarticular infections	18 (1.5%)	5
Neoplasms (out of 289 cases from 15 case-series where details are available)		
Lynphomas (including Hodgkin, Non-Hodgkin, not specified)	169 (58.5%)	9
Solid tumors (not specified)	25 (8.7%)	4
Leukemias	17 (5.9%)	7
Other cancers (not specified)	14 (4.8%)	5
Myelodysplastic syndrome	11 (3.8%)	7
Colon cancers	10 (3.5%)	5
Multiple mieloma	8 (2.8%)	6
Gastric cancers	5 (1.7%)	3
Mesotheliomas	5 (1.7%)	3
Castleman’s diseases	4 (1.4%)	3
NIIDs (out of 642 cases from 17 case-series where details are available)		
Adult-onset Still’s disease	177 (27. 6%)	15
Systemic Lupus Erythematosus	71 (11.1%)	14
Vasculitis	63 (9.8%)	8
Rheumatic Polymyalgia	44 (6.9%)	8
Giant Cells Arteritis	32 (5.0%)	6
Mixed connective diseases (not specified)	31 (4.8%)	5
Sarcoidosis	21 (3.3%	7
Rheumatoid Arthritis	17 (2.6%)	6
Wegener Granulomatosis	14 (2.2%)	4
Polyarteritis nodosa	13 (2.0%)	5

When compared with old case series, no differences emerged in the frequencies of infectious diseases and neoplasm, while NIIDs were significantly increased in the present analysis (Table 4). The percentage of FUO remaining without diagnosis tended to decrease in more recent case-series.

**Table 4:** Comparison of main diagnostic categories among FUO case-series in 2005–2015 and FUO case-series in 1995–2004

	Old case-series (1995–2004)	New case-series (2006–2014)	*p*-value
N° of patients	1488	3164	–
Male (%)	56,2	49,6	–
Mean age	40,6	45,8	–
Infectious Diseases	545 (37%)	1197 (38%)	0,428
Neoplasm	167 (11%)	366 (12%)	0,731
NIID	236 (16%)	661 (21%)	**< 0,001**
Others	155 (10%)	206 (7%)	**< 0,001**
No diagnosis	385 (26%)	734 (23%)	0,051

Variables with significant statistical association (*p* < 0,05) are in bold

### Factors influencing the distribution of final diagnosis

**Fig. 3:**
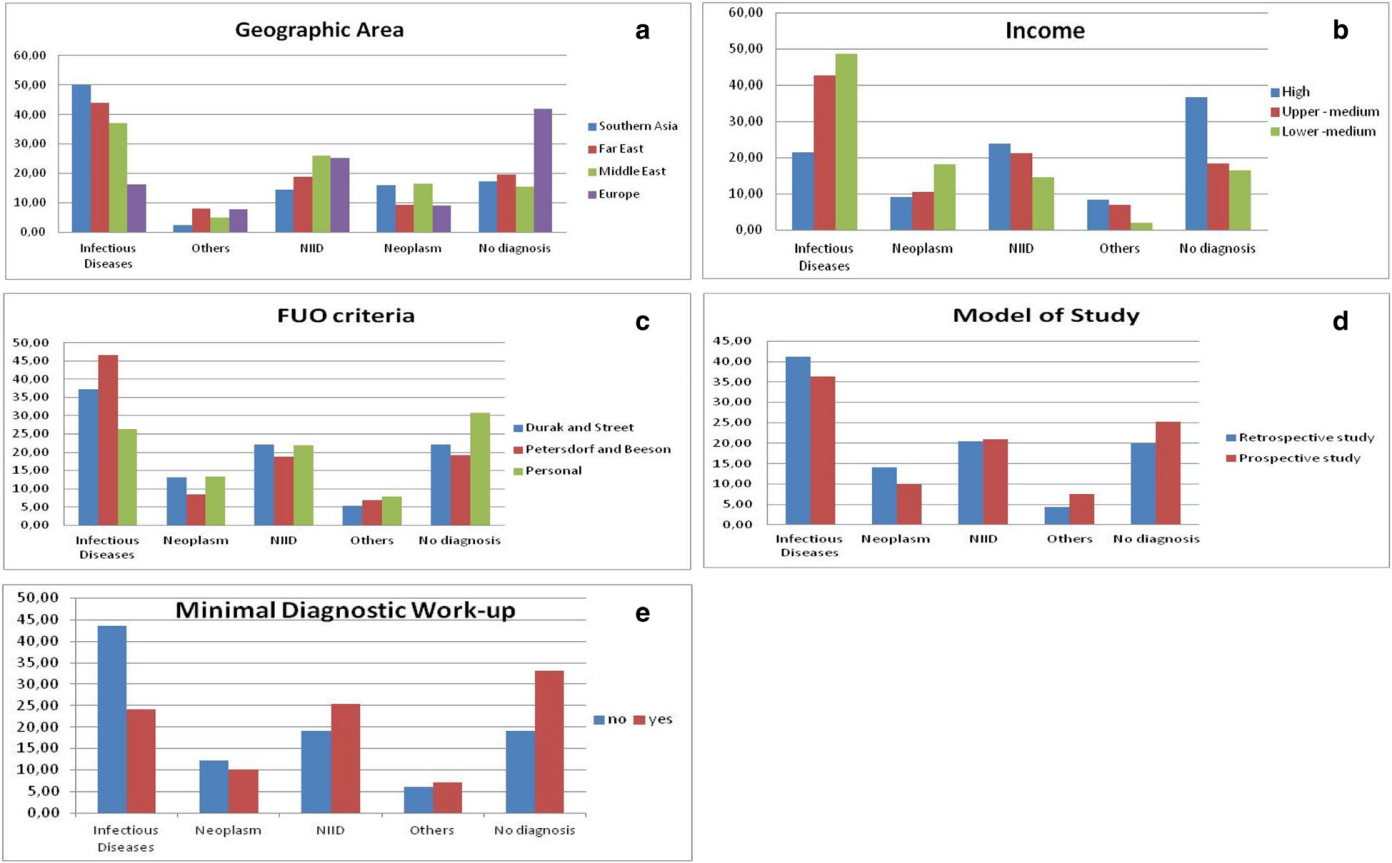
**a**-**e** Distribution of diagnostic categories associated to geographic area; income level of country, criteria used for FUO definition, model of study, and use of a Minimal Diagnostic Work-up to be performed before defining a fever as FUO

We investigated the association of different factors (geographic area, income of the country where the study was performed, model of study, FUO definition criteria, and routine adoption of a Minimal Diagnostic Work-up) with the diagnostic outcome (Fig. 3 a-e).

**Fig. 4:**
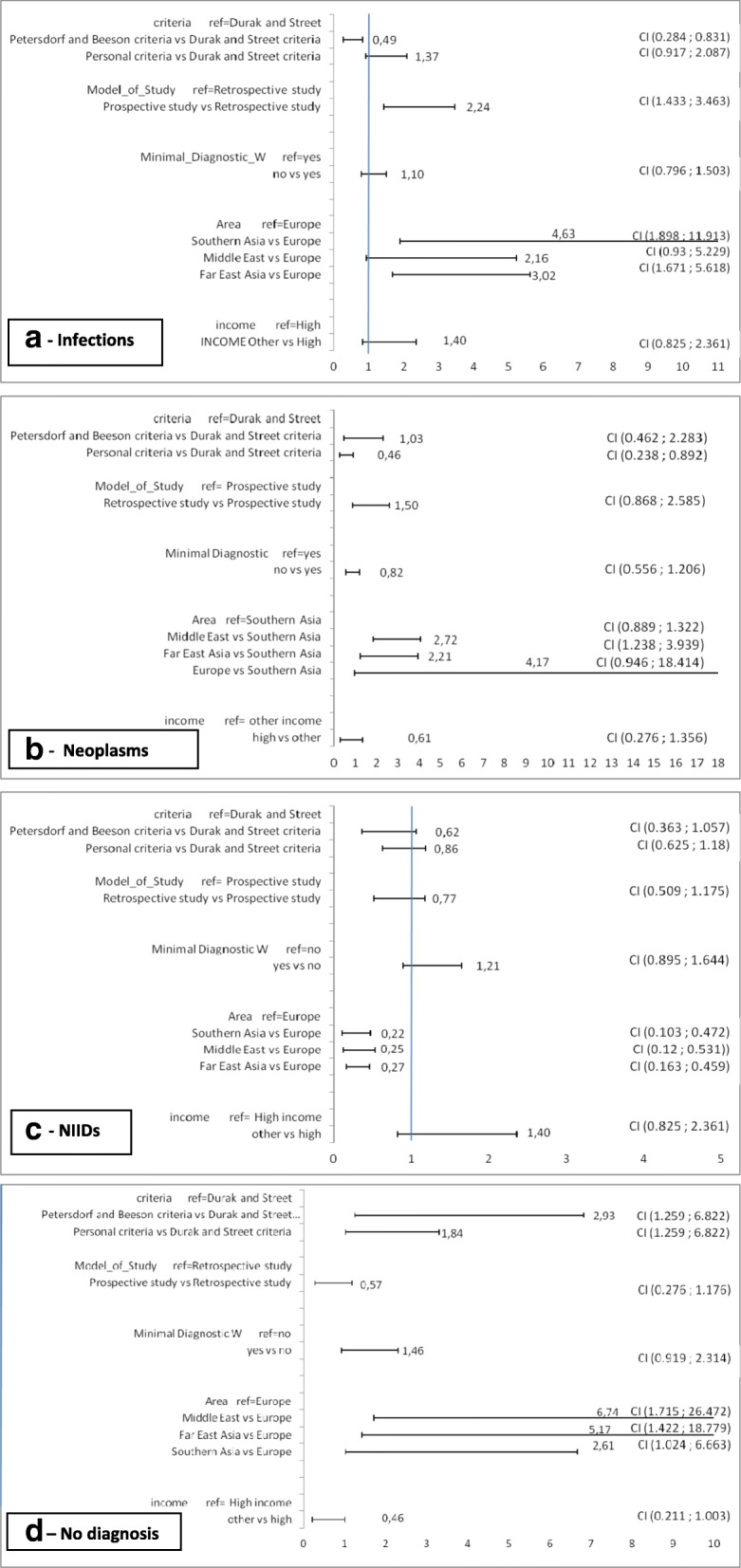
**a**-**d** Multivariate logistic regression models for infection (**a**), neoplasm (**b**), NIID (**c**), undiagnosed FUO (**d**)

At multivariable analysis (Fig. 4 a-d), all diagnostic outcomes were influenced by the geographical area where the study was performed: in particular, the risk of having an infection was more than 4 times higher in Southern Asia (OR 4.6; C.I. 1,89-11,91) and 3 times higher in Far East Asia (OR 3.0; C.I. 1,67-5,62) than in Europe; the diagnosis of NIID was more frequent in Middle East and Far East Asia than Southern Asia, the risk of having a NIID in Europe vs Southern Asia was close to significance (OR 4.17; IC 0.95–18.41); the diagnosis of neoplasm was more frequent in the case-series from Southern Asia and Middle East vs those from Far East Asia and Europe. The risk of having an undiagnosed FUO was higher in Europe vs all other geographical areas. The prospective model of the study was associated with a higher risk to have infections (OR 2,24; C.I. 1,43-3,46); the frequency of neoplasm was lower if Durak and Street criteria were used, and that of NIID if personal criteria were used. Among other factors analysed, the routine use of a Minimal Diagnostic work-up is not significant in any diagnostic categories, even if the performance of the work-up is associated with a lower prevalence of infections and a higher prevalence of undiagnosed FUO. Similarly, the income level of the country is not statistically significant, but in general there is an association between study country income level and ID and neoplasms (increasing when the income decreases) and NIID and undiagnosed FUO (increasing when the income increases),

## Discussion

The general interest towards FUO seems to have increased over time: the present analysis covering a study period from 2005 to 2015, included 18 studies and 3164 patients, as compared to 11 papers published and 1488 patients retrieved in our previous analysis [8]. These differences could be due to a major number of patients with fever who can access medical investigation and care. In fact, especially in lower-medium and upper-medium income countries, the urbanization and the general increase of quality and duration of life may have led to the detection of some feverish patients who would not get medical attention in a rural, poorer environment. At the same time, the increased number of patients may be due to a renewed interest into FUO, mainly in Asian lower-medium and upper-medium income countries.

Surprisingly, no case-series were available from the Americas and from Africa. In African countries, it may be due to the vast prevalence of acute febrile illness overwhelming the health systems, while we have no reasonable explanations for the lack of studies from North, Central and South America.

### Main study finding

Our approach based on income per region revealed a high heterogeneity in patient selection and particularly in diagnostic flowcharts. Infectious diseases remain the main cause of FUO. This is evident for lower-medium and upper-medium income countries, where infectious diseases represent 48 and 42% of the final diagnoses, respectively. Among infections, many “hard-to-diagnose” infectious diseases were included, such as mycobacterial diseases, endocarditis, internal abscesses, and osteoarticular infections. Possibly, recent advances in diagnostic imaging, such as the larger use of CT-scan, and in molecular biology laboratory methods contributed to these diagnoses.

NIIDs significantly increased over time, representing in new case-series 21% of final diagnoses. Advances in knowledge and clinicians’ awareness for these pathologies increased in recent decades, and this probably led to their higher prevalence among FUO. Another factor potentially contributing is the increased use of basic immunological tests in the minimal diagnostic work-up among the new case-series, that may have contributed to identify potential diagnostic clues about NIID among FUO patients. On the contrary, despite advances in diagnostic imaging and the diffusion of these methodologies to upper-medium and lower-medium income countries too, the prevalence of neoplasm did not change over time, such as the prevalence of FUO that remained undiagnosed.

A minimal diagnostic work-up, to be performed before qualifying a fever as a FUO, is not widely adopted. Considering the differences in diagnostic resources and capabilities in different geographic areas, the application of a merely quantitative criteria (3 days of in-hospital investigations or 3 outpatient visits) seems inadequate. Instead, the application of a minimal set of diagnostic procedures, including biochemistry, blood and urine cultures, basic imaging procedures and a set of infectious diseases screening tests determined on local epidemiological data, seems more reasonable, and has been advocated by some authors [3–6, 20, 25]. In this way, the patients classified as having FUO would be more easily comparable. However, despite these reasons, the use of a minimal diagnostic work-up is less frequent among new case-series, and mostly limited to studies from Europe or other high-income countries.

Mostly all explored factors influenced the final diagnostic outcomes, according to logistic regression. The geographic area of the study strongly influenced the distribution of final diagnoses: infections, as expected, were more frequently present in Southern and Far-East Asia; NIIDs were less frequently diagnosed in Southern Asia (case-series from India and Pakistan), where the clinical awareness towards these diseases is supposed to be lower; instead, do not have a clear geographic distribution. Undiagnosed FUO are disproportionally present in Europe: among the 4 European case-series, a minimal diagnostic work-up was applied in 3, and this might have contributed to select more challenging FUO cases, a sort of “real FUO”, consequently more difficult to be diagnosed. Of note, the use of diagnostic work-up, as “standing-alone factor”, is not associated with a higher prevalence of undiagnosed FUO at logistic regression.

### Limitations of the study

A possible limitation is represented by the great heterogeneity of the different studies, which may introduce some bias, using either multilevel or non-multilevel logistic regression analysis. Further studies on the same issue may overcome this limitation using Bayesian multilevel model that have shown potential to perform well with limited clusters in some scenarios [29, 30].

Another limitation might reside in the search criteria: we did not explore “grey literature”, including abstracts, reports, congress communications. Moreover, we limited our research to English language papers, and this may have led to the exclusion of papers written in other largely diffused languages, such as French and, especially, Spanish (and this may be the reason for the lack of studies from Central and South America). Other intrinsic limitations were the retrospective design of most studies and the different criteria used for including patients (Petersdorf and Beeson criteria, Durak and Street criteria, and personal criteria in some cases), leading to a poor comparability.

## Conclusions

Despite these limits, our study puts forward many interesting remarks. Even if infections represent the most frequent category among final diagnosis, our study confirms that the spectrum of FUO is huge, including many different diseases and conditions, and suggests that the proportion of FUO diagnosed as NIID has been increasing in recent decades. The presence of FUO that remain undiagnosed despite extensive investigations suggests the need for further research in this field. Of note, the prevalence of undiagnosed FUO is higher exactly where the most advanced diagnostics are available, suggesting the existence of diseases and conditions that are still elusive.

Strategies for optimizing the diagnostic approach for FUO should consider the main prevalent causes of fever in different areas, the local epidemiology, and the resources available. For these reasons, a global standardized diagnostic approach to FUO is not suitable. On the contrary, a standardization of FUO definition criteria is advisable. In particular, the adoption of a generally agreed minimal diagnostic work-up to be performed before qualifying a fever as FUO, would increase the generalisability and comparability of results of further studies.

In conclusion, despite the considerable advances in medical diagnostics and therapeutics, FUO still represent an intriguing challenge for clinicians, and further studies on this issue will continue to be advisable.

## Data Availability

All relevant data supporting the findings is contained within the manuscript. Database used for statistical calculations is available on request.

## Abbreviations

CMV: Citomegalovirus
FUO: Fever of Unknown Origin
HIV/AIDS: Human Immunodeficency Virus / Acquired Immunodeficency Syndrome
ID: Infectious Diseases
NIID: Non-infectious inflammatory diseases
PRISMA: Transparent Reporting of Systematic Reviews and Meta-Analyses
